# Clinical, hemispheric, and autonomic changes associated with use of closed-loop, allostatic neurotechnology by a case series of individuals with self-reported symptoms of post-traumatic stress

**DOI:** 10.1186/s12888-017-1299-x

**Published:** 2017-04-19

**Authors:** Charles H. Tegeler, Jared F. Cook, Catherine L. Tegeler, Joshua R. Hirsch, Hossam A. Shaltout, Sean L. Simpson, Brian C. Fidali, Lee Gerdes, Sung W. Lee

**Affiliations:** 1 Department of Neurology, Wake Forest School of Medicine, Medical Center Boulevard, Winston-Salem, NC 27157 USA; 2 Hypertension and Vascular Research Center, Wake Forest School of Medicine, Medical Center Boulevard, Winston-Salem, NC 27157 USA; 3 Department of Biostatistical Sciences, Wake Forest School of Medicine, Medical Center Boulevard, Winston-Salem, NC 27157 USA; 4 Brain State Technologies, 15150 North Hayden Road, Suite 106, Scottsdale, Arizona 85260 USA

**Keywords:** Post-traumatic stress, Hemispheric asymmetry, Temporal lobe, Insula, Brain electrical activity, Heart rate variability, Baroreflex sensitivity, RDoC, Neurotechnology, Allostasis

## Abstract

**Background:**

The objective of this pilot study was to explore the use of a closed-loop, allostatic, acoustic stimulation neurotechnology for individuals with self-reported symptoms of post-traumatic stress, as a potential means to impact symptomatology, temporal lobe high frequency asymmetry, heart rate variability (HRV), and baroreflex sensitivity (BRS).

**Methods:**

From a cohort of individuals participating in a naturalistic study to evaluate use of allostatic neurotechnology for diverse clinical conditions, a subset was identified who reported high scores on the Posttraumatic Stress Disorder Checklist (PCL). The intervention entailed a series of sessions wherein brain electrical activity was monitored noninvasively at high spectral resolutions, with software algorithms translating selected brain frequencies into acoustic stimuli (audible tones) that were delivered back to the user in real time, to support auto-calibration of neural oscillations. Participants completed symptom inventories before and after the intervention, and a subset underwent short-term blood pressure recordings for HRV and BRS. Changes in temporal lobe high frequency asymmetry were analyzed from baseline assessment through the first four sessions, and for the last four sessions.

**Results:**

Nineteen individuals (mean age 47, 11 women) were enrolled, and the majority also reported symptom scores that exceeded inventory thresholds for depression. They undertook a median of 16 sessions over 16.5 days, and 18 completed the number of sessions recommended. After the intervention, 89% of the completers reported clinically significant decreases in post-traumatic stress symptoms, indicated by a change of at least 10 points on the PCL. At a group level, individuals with either rightward (*n* = 7) or leftward (*n* = 7) dominant baseline asymmetry in temporal lobe high frequency (23–36 Hz) activity demonstrated statistically significant reductions in their asymmetry scores over the course of their first four sessions. For 12 individuals who underwent short-term blood pressure recordings, there were statistically significant increases in HRV in the time domain and BRS (Sequence Up). There were no adverse events.

**Conclusion:**

Closed-loop, allostatic neurotechnology for auto-calibration of neural oscillations appears promising as an innovative therapeutic strategy for individuals with symptoms of post-traumatic stress.

**Trials registration:**

ClinicalTrials.gov #NCT02709369, retrospectively registered on March 4, 2016.

## Background

By most measures, there is room for improvement in the healthcare of individuals with symptoms of post-traumatic stress. Expert consensus statements in the last decade have held that psychotherapies for posttraumatic stress disorder (PTSD), based on controlled re-exposure to the traumatic event, are the most evidence-based treatment [1, 2]. However there are persisting concerns about this modality due to high rates of non-response and dropout [3–5]. Moreover, sleep disturbance is a core feature of PTSD [6] and a predictor of worse global functionality [7], yet sleep improvement is typically elusive after completion of PTSD-specific behavioral therapies [8, 9]. While SSRI and other medication prescriptions are common, the overall evidence for the efficacy of psychopharmacological agents for individuals with PTSD is generally considered to be modest at best [1, 10]. Editorialists have affirmed that “New innovative and engaging approaches for the treatment of PTSD are needed” [11].

Recently, a bihemispheric autonomic model (BHAM) has been proposed as an integrative approach to understanding the effects of traumatic stress on health and behavior [12, 13]. The BHAM begins by recognizing that the right and left hemispheres have differential responsibility for management of efferent sympathetic and parasympathetic activity, respectively, and this division of labor is represented most prominently at the bilateral insular cortices [14], which are deep to the temporal lobes. The BHAM posits that traumatic stress (physical or emotional) can produce a risk for the development of dominant and maladaptive asymmetries of brain activity in homologous regions of the hemispheres (insulae and elsewhere) that are involved with downstream regulation of the autonomic nervous system (ANS). Specifically, the experience of an activating traumatic stress may increase the risk for rightward dominant asymmetry that produces an autonomic bias for sympathetic fight-flight physiology. Repeated stressors – or a single overwhelming stress – may increase the risk for leftward dominant asymmetry that produces a parasympathetic freeze state, which may be associated with emotional numbing or dissociation.

The model’s attention to lateralized cortical management of the ANS aligns with the transdiagnostic, dimensional, and brain-based perspective that is encouraged by the RDoC initiative [15], and this focus can integrate findings which might have otherwise unappreciated relationships. For example, the BHAM predicts the observation that PTSD is associated with a range of physical health disorders [16–18] including, effectively, accelerated physiological aging [19]. While the role of the ANS for mediating adverse behavioral and health states tends to be systematically under-recognized in clinical medicine [20], numerous researchers have used the metrics of heart rate variability (HRV) and baroreflex sensitivity (BRS), which are indicators of the capacity of the parasympathetic division to buffer sympathetic arousal, as a window on neural regulation of the cardiovascular system. Studies consistently show that HRV and BRS are diminished in patients with PTSD [21–25].

Furthermore, the BHAM encourages new thinking in the domain of therapeutic strategy. A thesis of the model is that relative symmetry in the activity of bilateral cerebral hemispheric regions responsible for management of the autonomic nervous system (i.e. insulae and other regions of temporal cortex) is likely to be characterized by a state of relative autonomic optimality, associated with relatively small and healthy fluctuations between leftward and rightward asymmetry of activity in those regions. A corollary is that there may be value to interventions that can facilitate brain activity to calibrate away from maladaptive forms of hemispheric asymmetry, toward symmetry. Attenuation of rightward dominance in temporal lobe high frequency asymmetry has been reported from use of allostatic neurotechnology in individuals with insomnia, with a trend for reduction in asymmetry score to be associated with reduction in symptoms of sleep disturbance [26].

The objective of the present study was to evaluate the effects of closed-loop, allostatic, acoustic stimulation neurotechnology on clinical symptoms, temporal lobe high frequency electrical asymmetry, and autonomic cardiovascular regulation (HRV and BRS), in a case series of individuals with self-reported symptoms of post-traumatic stress. We hypothesized that use of the intervention would be associated with decreased levels of symptomatology, decreased temporal lobe high frequency electrical asymmetry, and improvements in parasympathetic measures of autonomic cardiovascular regulation. Temporal lobe asymmetry was chosen because of predictions associated with the BHAM, and a high frequency range of electrical activity was chosen in consideration of potential associations between trauma, sleep disturbance, and increased activity in this range [27, 28]. Secondarily we explored for possible relationships between baseline temporal lobe high frequency asymmetry, symptom severity, and heart rate variability. Changes in insomnia symptomatology and neurophysiological hyperarousal demonstrated by these individuals are reported elsewhere.

## Methods

### Participants

Participants were drawn from an ongoing, open label, exploratory protocol whose objective has been to identify clinical cohorts that may benefit from use of allostatic neurotechnology. The study is a single site IRB-approved project, carried out in the Department of Neurology at Wake Forest School of Medicine in Winston-Salem, North Carolina, USA. Participants with one or more neurological, cardiovascular, or psychophysiological conditions have been enrolled through clinician referrals and informal networks, producing a relatively open sampling frame that can support the advance of transdiagnostic clinical neuroscience research [15]. Exclusions are physical disability to attend study visits, known seizure disorders, total hearing impairment bilaterally, or ongoing use of benzodiazepine, opiate or anti-psychotic medications. Of 152 participants enrolled in the open label study between August 11, 2011 and April 28, 2014, nineteen participants reported a history of exposure to a traumatic stressor and identified symptoms related to post-traumatic stress as the primary reason for enrollment, and they comprise the cohort for the present report. Individuals met DSM-IV-TR [29] symptom criteria for PTSD on the basis of self-reported scores on PCL-C domains, rating “moderately” or above to at least one “B” item (intrusive recollection), at least three “C” items (avoidant/numbing), and at least two “D” items (hyperarousal). All nineteen participants reported more than one month duration of symptoms.

### Neurotechnology-guided strategy for allostatic auto-calibration of neural oscillations

The intervention entailed use of a closed-loop, allostatic acoustic stimulation neurotechnology, intended to support auto-calibration of neural oscillations (High-resolution, relational, resonance-based, electroencephalic mirroring, or HIRREM® [26]; Brain State Technologies, Scottsdale, Arizona). HIRREM entails recording of brain electrical activity from scalp-based sensors, computer-guided algorithmic analysis of neural oscillatory frequencies for translation into sonic frequencies, and the return of acoustic signals back to the brain in real time (through the peripheral auditory nervous system). Procedures for the intervention have been discussed in detail previously [26]. Each participant began with a baseline assessment (45 min) to evaluate patterns of activity in the brain electrical frequency spectrum with respect to hemispheric symmetry, and ratios of energy across frequencies ranging from 0 to 48 Hz. The assessment consisted of two-channel recordings of brain electrical activity from at least six paired locations on the scalp (F3/F4, C3/C4, T3/T4, P3/P4, O1/O2, FZ/OZ; referenced to ears, linked), with the recipient at rest and while carrying out a task, using sensors and amplifiers (Brain State Technologies, Scottsdale, Arizona) that sample at 256 Hz. Recordings were conducted at each location with eyes closed (one minute), eyes partially open to permit transition in arousal state (one minute), and eyes open (one minute while performing a mental task, e.g. recalling numbers, reading a passage, etc.). Trained technologists evaluated assessment data to choose protocols for the initial intervention session. Protocols consisted of recording brain electrical activity through generally two channels, with sensors placed at homologous regions of the hemispheres (F3/F4, C3/C4, T3/T4, P3/P4, O1/O2, Fp1/Fp2) and also other locations depending on the judgment of the technologist. In real time, software algorithms analyzed specific ranges of the brain electrical frequency spectrum, identified dominant frequencies on the basis of proprietary mathematical formulae, and translated those frequencies to acoustic stimuli (audible tones of variable pitch and timing) which were presented to participants through standard earphones (Creative EP-630 or Sony Stereo Headphones MDR-EX58V) with as little as an eight millisecond delay. Volume (decibels) of acoustic stimulation was adjusted by each participant in accordance with their preference. The initial and subsequent sessions (approximately 90 min each) consisted of four to ten protocols (five to forty minutes each), some done with eyes closed and some with eyes open, with the participant being asked to relax while sitting or reclining comfortably in a zero-gravity chair. Specific protocols for successive sessions were chosen based on brain electrical data from the preceding session, which for purposes of technologist review was aggregated in broad-band frequency ranges (Hz; <1.0, 1.0–3.0, 3.0–5.5, 5.5–7.5, 7.5–10.0, 10.0–12.0, 12.0–15.0, 15.0–23.0, 23.0–36.0, 36.0–48.0). Brain regions were chosen with special attention to patterns of activity suggestive of dominant hemispheric asymmetries and/or suboptimal ratios of energy across the frequency spectrum [26]. Algorithms are designed to support de-establishment of relatively invariant and potentially maladaptive activity patterns. Participants generally completed two sessions in a half day, separated by a 20–30 min break. The decision for the total number of sessions to be received was based on impressions of clinical improvement or plateau, including evaluation of the participants’ brain pattern evolution over the course of their sessions, as well as the participants’ schedules and preferences. All participants continued with their usual medical or behavioral care. As a closed-loop strategy [30], HIRREM is explicitly designed to depend on brain activity as such, as opposed to the volition or learning skills of the client. Furthermore it is aligned with the *allostasis* model of physiological regulation, which recognizes the significance of neural regulation across organ systems, and the dependence of biological system functionality on the changing features of the local context [31–34].

### Measures of temporal lobe high frequency electrical asymmetry

One-minute epochs of brain electrical activity data from bilateral temporal lobes (T3 and T4 locations in the 10–20 International EEG system) were collected from the assessment and a temporal lobe exercise from each of the first four and final four sessions, in order to permit comparisons of activity from the early and latter phases of the intervention, for individuals who had differing numbers of total sessions. For each session, two epochs were analyzed, the first and the penultimate minute of the session. The penultimate minute was used to avoid potential recording artifacts related to completion of the final seconds of a given exercise (e.g. muscular contractions as participants became more alert). Tracings of amplitudes in ten frequency ranges (see Section 2.2) were visually inspected for sustained spikes or drops in amplitudes and cross-referenced with technologist session notes, to remove data that were considered to be potentially indicative of sensor displacement. Temporal lobe high frequency (23–36 Hz) asymmetry scores were calculated for each epoch by subtracting average amplitude at T3 from average amplitude at T4 and dividing by the lesser of the two. A positive asymmetry score indicates T4-dominance and a negative asymmetry score T3-dominance. On the basis of an a priori hypothesis about the significance of rightward versus leftward asymmetry in temporal lobe electrical activity [12], participants were divided into three groups according to their asymmetry scores at baseline assessment – T4 dominant (≥10%), T3 dominant (≤ − 10%), or balanced (absolute value < 10%). For each dominance group, change in asymmetry during the initial and latter stages of the intervention was evaluated in an exploratory way as follows. The slope of a fitted line was determined over the initial assessment and first four sessions, and the last four sessions, based on the median asymmetry scores over the course of those sessions. In order to assess whether these fitted lines reflected a tendency for change in asymmetry score, indicated as a positive or negative slope, a one-sample linear regression t-test was performed on each slope, with the null hypothesis being that it was zero. It should be noted that although this technique is common for interpreting the significance of a trend line, the underlying data consisted of median values for asymmetry scores and entailed intra-subject correlations, and thus the probability of randomness (*p* value) generated by this statistical test is best considered exploratory. Statistical tests were performed using SAS software version 9.3 (SAS Institute Inc., Cary, North Carolina, USA).

### Symptom inventories for post-traumatic stress and depression

The Posttraumatic Stress Disorder Checklist (PCL-C; [35]) measures symptoms that may pertain to any traumatic life experience, and includes PTSD criteria B, C, & D measures from the DSM-IV-TR. Those with prior military service also completed the military version (PCL-M), which was used for reporting in the present paper. Seventeen items are rated on a Likert scale with a composite score range of 17–85, and a score of 44 or greater indicates a high likelihood for PTSD diagnosis [36]. A decrease of 10 points or greater in the PCL-C is considered a clinically significant improvement [37], and group-level change in PCL score was also analyzed through a paired t-test. Symptoms of depressive mood were evaluated by either the Center for Epidemiologic Studies Depression Scale (CES-D; [38]), or the Beck Depression Inventory (BDI; [39]). CESD scores range from 0 to 60, with a score of 16 or greater commonly used to indicate risk for clinically relevant depressive symptomatology. BDI scores range from 0 to 63, yielding classification of minimal depression (0–13), mild depression (14–19), moderate depression (20–28), and severe depression (29–63; [40]), and a score of 14 or greater was used as the threshold value. Post-HIRREM symptom data were collected within two weeks of the final HIRREM session.

### Assessment of heart rate variability and baroreflex sensitivity

For twelve subjects, short-term (5 min, 1000 Hz) continuous blood pressure and heart rate recordings (BIOPAC, Santa Barbara, CA) were collected under conditions of spontaneous breathing, within thirty minutes prior to the baseline HIRREM assessment, and within two weeks after completing the final HIRREM session. Heart rate variability (HRV) was assessed as the standard deviation of the NN interval (SDNN) in the time domain and as the absolute values of the low and high frequency components. Baroreflex sensitivity (BRS) was assessed by the sequence method (Nevrokard BRS, Nevrokard Kiauta, Izola, Slovenia). Measures of autonomic cardiovascular regulation are presented as pre and post-HIRREM values and percentage differences, and comparisons were made using paired t-tests.

### Relationships between temporal lobe high frequency asymmetry, heart rate variability, and clinical symptoms

Exploratory analysis was performed to detect possible relationships between temporal lobe high frequency electrical asymmetry and symptom severity or heart rate variability (SDNN). Each individual’s baseline SDNN value (when available) was plotted as a function of the temporal lobe high frequency asymmetry score from their HIRREM assessment, using a second-order polynomial regression equation. Similar analyses were conducted to detect possible relationships between temporal lobe high frequency asymmetry and the symptom inventory scores.

## Results

Participants’ baseline characteristics, number of HIRREM sessions received, and scores on the PCL before and after the intervention are shown in Table 1. Mean age was 47 years (*SD* 14.9), 58% were women, and they reported a median of 3.5 years (range 3 months to 50 years) of symptoms after a traumatic event. All reported a history of multiple health conditions. 18 subjects provided both pre- and post-intervention data for the PCL and CES-D/BDI inventories. At baseline 95% reported symptom levels for depressive mood that exceeded the threshold for potential clinical significance. Participants received a median of 16 (range 6 to 26) HIRREM sessions over a median of 16.5 days (range 9 to 184), with a median of 8 days (range 5 to 14) of visits to the office to receive sessions. One participant elected to discontinue before the follow-up data collection visit, citing a lack of motivation and a preference to resume a previously discontinued medication regimen. After the intervention, 89% reported decreases of at least 10 points on the PCL, and as a group the mean change in their PCL scores was −24.1 (*p* < .0001). 33% exceeded the threshold values for depressive symptomatology. There were no adverse events.

**Table 1:** Gender, self-reported health conditions, and clinical course of study participants

Gender	Self-reported health conditions	Total number of sessions	Days in office/Total days start to finish	Pre-Intervention PCL score	Post-Intervention PCL score
M	Hypertension, anxiety, depression, insomnia, ADHD	11	6/10	57	28
F	Acutaneous lupus, anxiety, insomnia	17	14/39	67	33
M	Hypertension, headaches, depression, anxiety, insomnia, ADHD	12	8/11	63	53
M*	Anxiety, depression, insomnia	14	8/15	47^a^	22^a^
F	Bipolar disorder, insomnia	11	6/9	59	39
F	Depression, insomnia	26	13/184	56	28
F	Migraine, insomnia	6	5/9	53	n/a
M*	Traumatic brain injury, migraines, insomnia	22	12/13	76	58
F	Anxiety, migraines, insomnia	16	8/25	73	31
M*	Traumatic brain injury, migraines, depression, anxiety, insomnia	17	10/18	55	53
F	Traumatic brain injury, Headaches, fibromyalgia, insomnia	17	9/25	77	43
F	Fibromyalgia, headaches, depression, hot flashes	16	8/26	51	32
M*	Traumatic brain injury, insomnia, ADHD	12	6/8	71	21
F	Traumatic brain injury, chronic pain, migraine, tinnitus, depression, insomnia	16	8/10	81	76
F	Hypothyroidism, seasonal affective disorder, hot flashes, anxiety, insomnia	19	11/26	51	24
M*	Traumatic brain injury, headaches, tinnitus, anxiety, insomnia	16	8/10	48	30
F	Hyperlipidemia, tinnitus, anxiety, insomnia, depression, ADHD, hot flashes	13	8/12	47	19
M*	Hypertension, hyperlipidemia, diabetes, cancer, chronic pain, tinnitus, anxiety, insomnia	18	10/59	40	28
F	Headaches, anxiety	16	11/39	58	26

*Trauma related to prior military service, for whom symptom changes are reported using PCL-M

^a^Scores are based on PCL-C

**Fig. 1:**
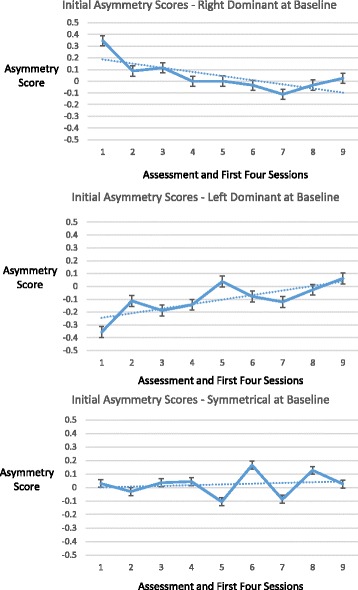
Initial scores for temporal lobe high frequency (23–36 Hz) asymmetry. Panels show median asymmetry scores for baseline assessment (hashmark 1 on *horizontal axis*) through the first four sessions (first and penultimate minutes of each session, hashmarks 2 through 9). *Vertical axes* indicate asymmetry scores, with positive and negative values indicating *rightward* and *leftward* asymmetry, respectively. *Top panel* shows data for participants whose baseline temporal lobe high frequency asymmetry was 10% or greater toward the *right* (*n* = 7); *middle panel* shows those whose baseline asymmetry was 10% or greater toward the *left* (*n* = 7); *bottom* shows those whose baseline asymmetry was less than 10% in either direction (*n* = 5)

**Fig. 2:**
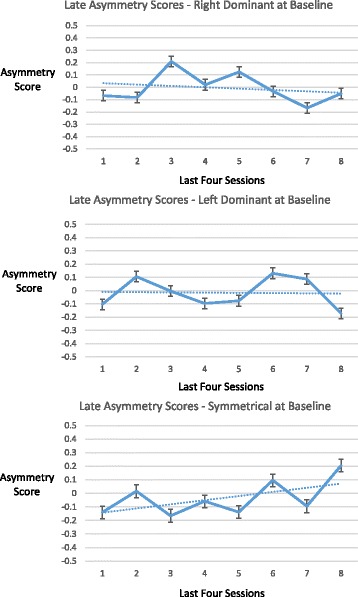
Later scores for temporal lobe high frequency (23–36 Hz) asymmetry. Panels show median asymmetry scores for the last four sessions (first and penultimate minutes of each session, hashmarks 1 through 8). See Fig. 1 legend for explanation of other elements

**Fig. 3:**
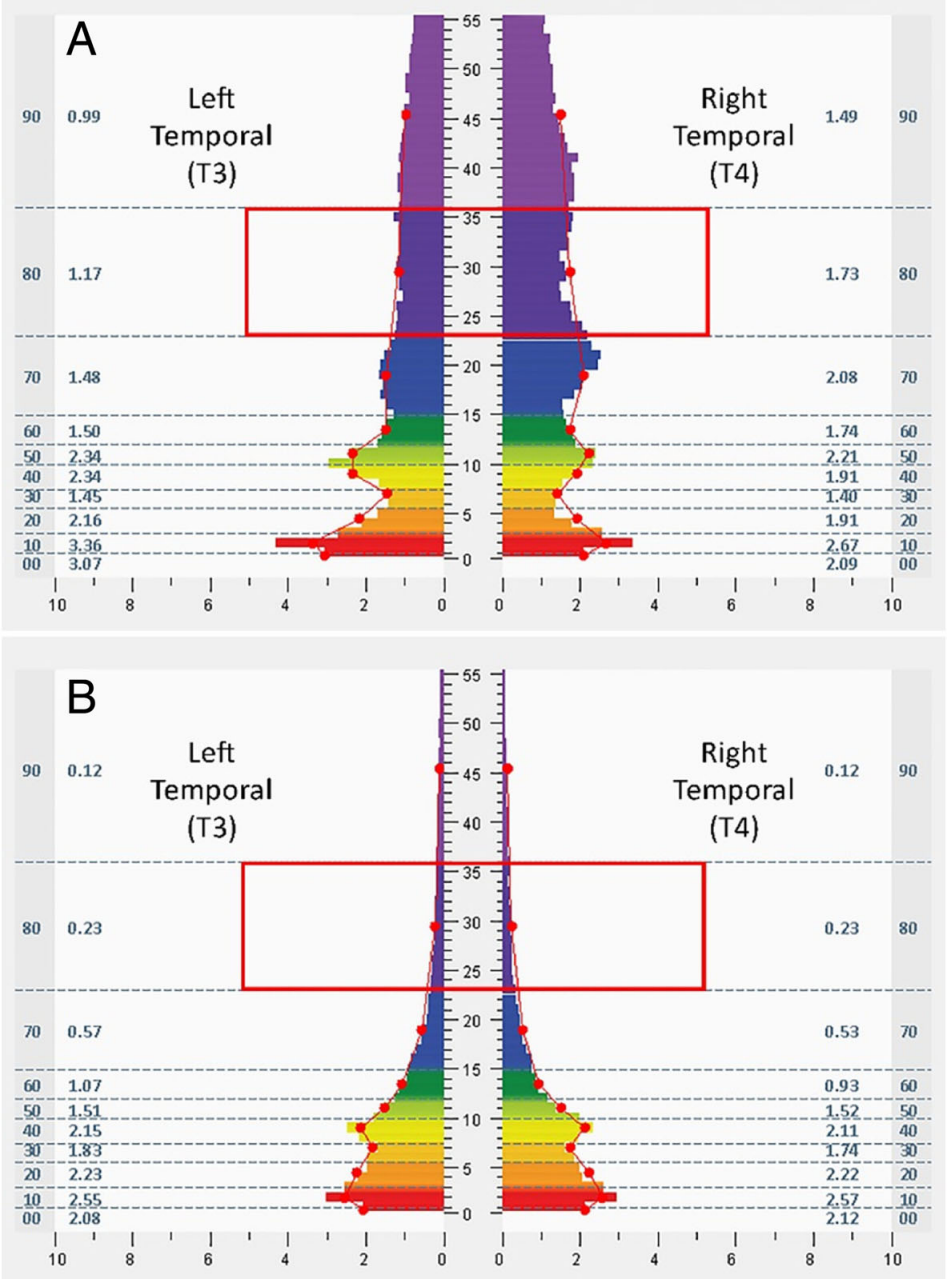
Spectrographs of bilateral temporal lobe electrical activity for a 71 year old male Vietnam War veteran with PTSD and multiple co-morbidities, from baseline assessment (**a**) and penultimate minute of the fourth HIRREM session (**b**). Data were collected from bilateral temporal lobes (T3 and T4 in the 10–20 International EEG system), with frequency (Hz, *vertical axis*) plotted against amplitude (microvolts, μv, *horizontal axis*). Individual color bars reflect amplitude averages for one minute of recording, eyes closed, at rest, without stimulation. Columns to the *left* and *right* of the color bars denote ten frequency ranges of aggregated data (Hz, 00: <1.0; 10: 1.0–3.0; 20: 3.0–5.5; 30: 5.5–7.5; 40: 7.5–10.0; 50: 10.0–12.0; 60: 12.0–15.0; 70: 15.0–23.0; 80: 23.0–36.0; 90: 36.0–48.0) and numerical values for averages in those ranges

**Fig. 4:**
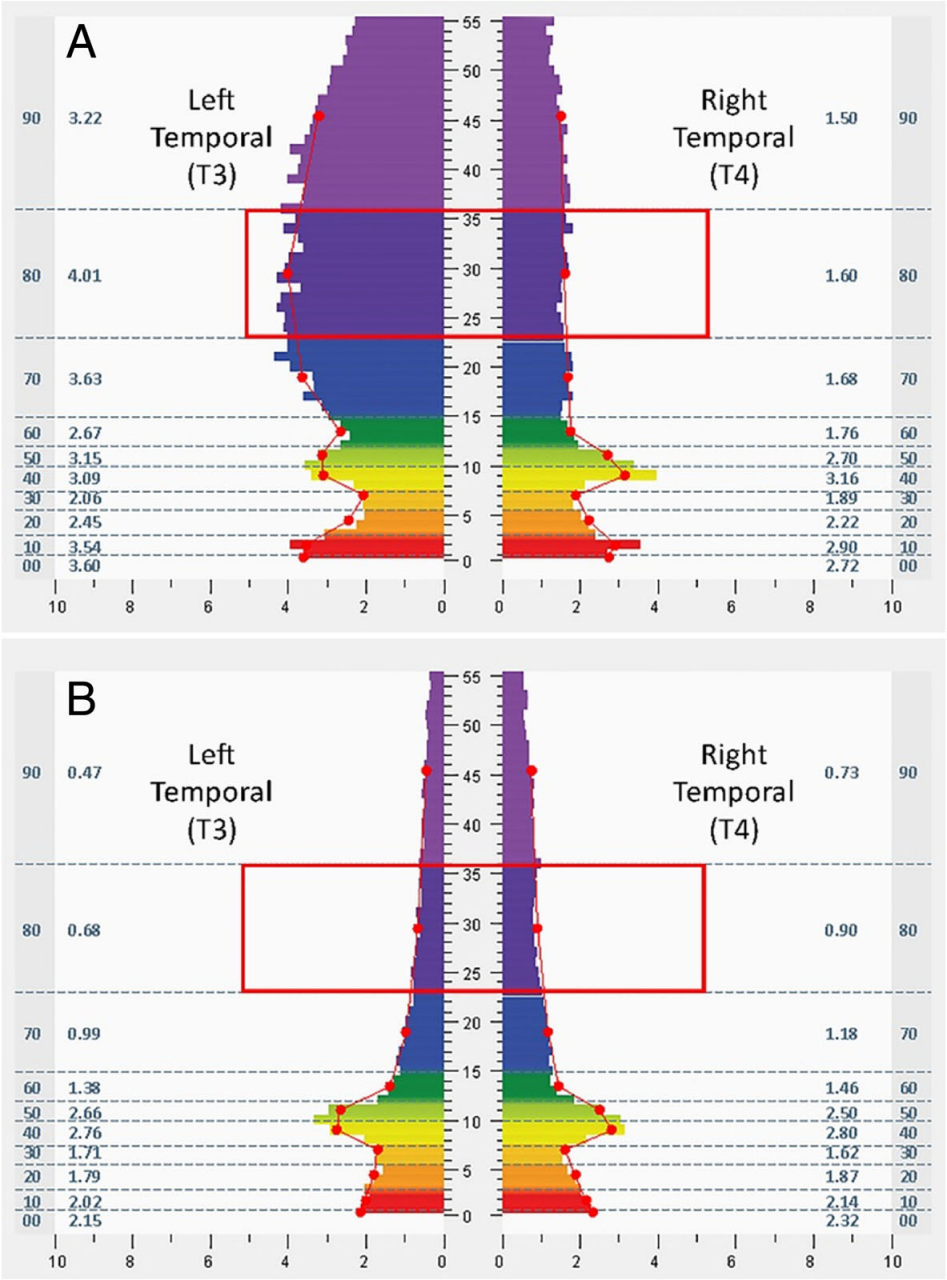
Spectrographs of bilateral temporal lobe electrical activity for a 50 year old woman with a history PTSD, migraine, and insomnia, from baseline assessment (**a**) and penultimate minute of the fourth HIRREM session (**b**). See Fig. 3 legend for detailed description of data elements

Changes in temporal lobe high frequency (23–36 Hz) electrical asymmetry are shown in Figs. 1 and 2. For the group of individuals who were rightward (T4) dominant (≥10%) at baseline, the slope of the trend line from the assessment through the first four sessions was negative (*p* = .023), indicating movement toward greater symmetry. Conversely, the group of individuals who were leftward (T3) dominant (≤ − 10%) at baseline demonstrated a positive slope in the trend line (*p* = .015) for their early sessions, also indicating movement toward greater symmetry. No statistically significant change in the asymmetry scores was shown for the group that did not have dominant asymmetry at baseline (<10% in either direction) over their early sessions, or for any of the groups over their final four sessions. For illustrative purposes, bilateral temporal lobe spectrographs for activity at baseline and during the penultimate minute of the fourth HIRREM session are shown for individuals whose baseline asymmetry was rightward (Fig. 3) and leftward (Fig. 4).

**Fig. 5:**
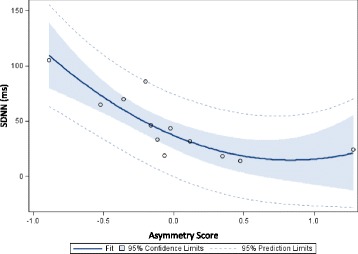
Fit plot of heart rate variability (*vertical axis*) versus temporal lobe high frequency asymmetry score (*horizontal axis*), at baseline. Data are from 12 individuals who underwent short-term blood pressure recordings prior to beginning use of allostatic neurotechnology. SDNN = standard deviation of the NN interval. Asymmetry scores greater than zero indicate *rightward* asymmetry, less than zero indicate *leftward* asymmetry

For the twelve participants who underwent short-term blood pressure recordings before and after HIRREM, values for measures of autonomic cardiovascular regulation are shown in Table 2. There were statistically significant increases in heart rate variability as measured in the time domain (SDNN) and baroreflex sensitivity (Sequence Up – indicative of lengthening of the RR interval with increasing blood pressure). Exploratory analysis demonstrated a significant negative correlation between rightward temporal lobe high frequency asymmetry at baseline assessment and SDNN (F-test *p* = .0014), as shown in Fig. 5. There was no statistically significant relationship between temporal lobe high frequency asymmetry and symptom scores for post-traumatic stress or depression (data not shown).

**Table 2:** Changes in measures of autonomic cardiovascular regulation after use of closed-loop allostatic neurotechnology

	Mean value at baseline (*SD*)	Mean value after HIRREM (*SD*)	*P* value
BRS Sequence Up [ms/mmHg]	13.2 (11.0)	17.3 (12.6)	0.009
BRS Sequence Down [ms/mmHg]	14.5 (9.6)	17.5 (17.8)	>0.2
BRS Sequence All [ms/mmHg]	13.9 (9.7)	18.0 (15.8)	>0.2
SDNN [ms]	46.2 (29.2)	53.4 (32.0)	0.026
LF [ms^2^]	1407 (2094)	2100 (3702)	>0.2
HF [ms^2^]	881 (1778)	1048 (2510)	>0.2
MAP [mmHg]	96.8 (4.5)	94.3 (9.8)	>0.2
HR [bpm]	63.3 (11.3)	65.3 (10.4)	>0.2

*BRS* Baroreflex sensitivity, *SDNN* Standard deviation of the normal beat-to-beat interval, *LF* Low Frequency, *HF* High Frequency, *MAP* Mean arterial pressure, *HR* Heart rate

## Discussion

In this case series, individuals with self-reported symptoms of post-traumatic stress undertook use of an allostatic neurotechnology for closed-loop auto-calibration of neural oscillations. After completing a series of sessions, the majority of participants reported clinically significant reductions in their symptoms of post-traumatic stress and depressive mood. Analysis of high-frequency (23–36 Hz) brain electrical activity patterns at the bilateral temporal lobes showed that at a group level, those who had either rightward or leftward asymmetry at their baseline assessment became more symmetrical over their first four sessions. In a subset of the participants for whom short-term blood pressure recordings were collected, statistically significant improvements were demonstrated in HRV in the time domain as well as BRS, suggesting increased parasympathetic capacity. The intervention was well tolerated, with 18 of 19 participants completing their course of sessions as recommended by the provider team.

The present findings are consistent with earlier studies reporting reductions in temporal lobe high frequency asymmetry, improvements in heart rate variability, or both, in the course of allostatic neurotechnology usage by adults with insomnia [41], adolescents with postural orthostatic tachycardia syndrome (POTS; [42]), and athletes with persisting post-concussion symptoms [43]. Other neurotechnologies proposed for treatment of PTSD include stimulatory techniques such as transcranial magnetic stimulation (TMS; [44]) and transcranial direct current stimulation (TDCS; [45]), which are open-loop approaches in that they do not record brain activity before delivering successive cycles of their intervention. EEG operant conditioning [46–48] is a form of reward-based training to achieve conscious self-management of brain rhythms (“learner-in-the-loop”), and a recent study found that receiving 40 sessions was associated with a significant but incomplete degree of PTSD symptom reduction [48].

The negative correlation at baseline between rightward temporal high frequency asymmetry and HRV is consistent with a report on the larger and more heterogeneous sample from which this case series was drawn [49], which contended that temporal lobe high frequency electrical asymmetry holds promise as a physiological unit of analysis for the RDoC domain of arousal. In contrast, the absence of a relationship between this metric and post-traumatic stress symptom severity gives preliminary indication that it is unlikely to be sufficient as a stand-alone biomarker of clinical PTSD status. This assessment is tempered by recognition that the participants had numerous comorbid health conditions which may have had independent effects on their asymmetry patterns. Others have reported that state-dependent brain electrical asymmetry during trauma-relevant stimulation is more likely than trait asymmetry under neutral conditions, to identify individuals with PTSD [50, 51].

Prima facie, we interpret the reduction in temporal lobe high frequency asymmetry over the first four sessions as a reflection of auto-calibration of neural oscillations [26]. In conjunction with the increased HRV and BRS that were demonstrated after completion of the sessions, the movement toward symmetry is consistent with a thesis of the BHAM that was noted in the Introduction. Increasing symmetry in temporal lobe high frequency electrical activity may have represented enhanced, centrally-directed, parasympathetic modulation of sympathetic arousal. If an effect on the core biology of autonomic regulation is shown to be durable and replicable in future studies, it would represent a genuine therapeutic advance. To date, there have been reports of improved HRV in patients with PTSD in association with usage of fluoxetine [52], behavioral therapy [53], HRV biofeedback [54], and eye movement desensitization and reprocessing [55]; other recent studies in military service members with PTSD have found that mindfulness meditation had no impact on HRV [56], and escitalopram was associated with HRV reduction [57].

An important limitation to the present study derives from its case series design and the absence of a control group. Above all, it could be conjectured that the results were primarily due to subjective expectation for benefit, positive provider-client interactions, regression to the mean, natural history of disease, or a combination of these factors. Nonetheless the physiological changes in temporal lobe electrical asymmetry and autonomic cardiovascular regulation tend to stand against this explanation, considering that placebo interventions in clinical research are less likely to impact objective outcome measures [58]. An additional limitation of the study is that the participant selection was based on their self-reported history and score on the PCL, and did not include a clinician assessment.

## Conclusion

In conclusion, this study found that individuals with self-reported symptoms of post-traumatic stress who undertook use of a closed-loop, allostatic, acoustic stimulation neurotechnology, reported significant reductions in clinical symptomatology that were accompanied by reductions in temporal lobe high frequency electrical asymmetry, and increased HRV and BRS. These improvements, in conjunction with the absence of adverse events or significant drop-outs, suggest that neurotechnology-assisted auto-calibration of neural oscillations holds promise as an innovative therapeutic strategy for individuals with symptoms related to post-traumatic stress.
